# Chinese smokers’ behavioral response toward cigarette price: individual and regional correlates

**DOI:** 10.1186/s12971-016-0078-7

**Published:** 2016-04-05

**Authors:** Tingzhong Yang, Sihui Peng, Lingwei Yu, Shuhan Jiang, William B. Stroub, Randall R. Cottrell, Ian R. H. Rockett

**Affiliations:** Center for Tobacco Control Research, Zhejiang University School of Medicine, Yuhangtang Road, Hangzhou, 310058 China; Health Services Administration Program, University of Evansville, Evansville, IN 47722 USA; Public Health Studies Program, School of Health and Applied Human Sciences, University of North Carolina, Wilmington, NC 28403 USA; Department of Epidemiology, School of Public Health, and Injury Control Research Center, West Virginia University, Morgantown, WV 26506-9190 USA

**Keywords:** Smoking, Behavioral response, Public education

## Abstract

**Background:**

Many studies have explored smokers’ behavioral response to cigarette prices at the individual level, but none have factored in regional variation and determinants. This study addresses these research gaps in the Chinese context.

**Methods:**

A cross-sectional multistage sampling process was used to recruit participants in 21 cities in China. Individual-level information was collected using standardized questionnaires. City-level variables were retrieved from a nationall database. Multilevel logistic regression analysis was used to assess price sensitivity variation at both individual and city levels.

**Results:**

Among 5660 current smokers, 5.9 % used non-self-paying cigarettes, 32.8 % purchased cigarettes in cartons, and 5.2 % decreased their smoking expenditure due to price. Multilevel analysis showed that individual demographic and smoking expenditure characteristics and regional smoking restrictions in work places, cigarette production, and media news coverage are associated with price sensitivity.

**Conclusions:**

This study adds substantially to the understanding of Chinese smokers’ behavioral responses to cigarette prices. Increasing smoker sensitivity to cigarette prices will require stronger tobacco control and public education campaigns.

## Background

Many studies have found that cigarettes price increases can reduce cigarette consumption [1–4]. Furthermore, the price elasticity of cigarette consumption in less developed countries is greater than of their developed counterparts [2, 3]. Several authors also analyzed price elasticity of cigarette consumers in China, but the results of these studies vary widely. Hu and Mao, Bishop et al. suggest people in China are sensitive to the price of cigarettes [2, 5], while Lance and Huang found that they are less sensitive to the price of cigarettes [6, 7], even if comparing with other low-income and middle-income countries [7]. In any case price elasticity of cigarette is closely related to the consumers’ behavioral response of the cigarette price. This is particularly important for understanding smokers’ cigarette consumption behavior and considering for the design and implementation of interventions to increase smokers’ sensitivity to the cigarette price.

Ecological models emphasize behavioral events are influenced by both individual and environmental variables. Understanding environmental influences on the price behavioral response is important from a public health perspective, particularly for formulating policy and designing and implementing effective interventions that take account of both environmental and individual influences on the behavior [8]. Furthermore, combing individual with regional level data can avoid ecologic and atomistic fallacies, which thus allows for separation of individual and contextual effects upon the behavior [5, 9]. Many studies explored smokers’ behavioral response to the cigarette price and individual level influences [2–9], but no studies included regional variation and determinants. This is probably because most studies have been confined to local and community subpopulations, and therefore have had no basis for analyzing regional variation in the price behavior response [2, 4]. China mainland has a vast territory, cultural diversity, large differences in economic and social development. By utilizing a large-scale, national population sample it is possible to conduct this study. This study will examine explanatory variables of smokers’ behavioral response toward cigarettes prices at both the individual and regional level. We hypothesized demographic characteristics, income, smoking expenditure and purchasing behaviors were associated with the price behavioral response. We are particularly concerned about influences of environmental smoking bans, cigarettes production, and media news coverage on price behavioral response. Many studies have found environmental smoking restrictions, cigarette production, and media news coverage was associated with smoking behaviors [1, 10–12]. We hypothesized that these variables may influence people’s belief, awareness, and attitudes about smoking and tobacco control, and further may affect the price behavioral response.

## Methods

### Study area and participants

This study employed a cross-sectional multistage sampling design. In Stage 1, 21 cities were selected from across China and differentiated by regional location. Nine were located in the east, five in the central region, and the remaining seven in the west, and these 21 cities are located account for 68 % of all provinces in China. Stage 2 involved selection of residential districts within each city. Two residential districts were randomly selected in the main urban zone of each city, excluding new building districts and subdistricts. In Stage 3, four communities were randomly selected within each residential district. In Stage 4, a family household registration (“hukou”) list was used to randomly sample households in each community. All individuals aged 15 years and older, who were permanent residents in these cities, were identified within each household. Individuals aged 15 years and older, who had lived in their home for at least a year, were identified within each household [13, 14]. Finally, one eligible participant was randomly selected from each family, with eligibility being determined by birthdate closest to the contact date [13, 14].

### Data collection

A self-administered questionnaire was scheduled, once an individual was identified and agreed to participate in the survey. Field staffs were fourth-year medical students from a local medical college, who had received a one-day training on the study protocol and interviewing procedures. The questionnaire was administered privately in participants’ homes or in a designated quiet place, such as a backyard or community park. Survey were conducted on Saturdays, Sundays, weekday evenings or other times when participants were available. After receiving oral instructions by staff about the survey and questionnaire, the questionnaire was given to each participant to complete. Completion took approximately 30 min. Participants could ask staff if they were confused about any items on the questionnaire. Each participant was given an opportunity to clarify confusing questions and adequate time to questionnaire completion. Participants were requested to resolve any omissions, as appropriate, and were given a token of appreciation (toothbrush and toothpaste, and other small gifts valued at approximately US$ 1.00) following questionnaire completion. The data were conducted between June and September in 2011.

A common research protocol was utilized across all 21 cities to assure homogeneity of interview and data collection. The study was approved by the Ethics Committee at the Medical Center, Zhejiang University, and verbal consent was obtained from all participants prior to data collection. Our methods have been extensively employed in smoking research in China, and they possess acceptable validity [13–16].

### Dependent variables

The price behavioral response was measured by smoking behavior change related to cigarette price, the following question was asked: “Are you smoking less because current cigarette prices are too high?” Response options were No/decrease a little/decrease some or more.

### Individual level variables

Sociodemographic characteristics: Age, gender, ethnicity, educational level, and occupation, income were included in this study.

Smoking expenditure and purchasing behaviors: Smoking expenditure status included frequency and quantity of smoking, smoking history, and smoking situation and were assessed through self-report. We employed standard methods for computing these measures [14, 16]. A current smoker was defined as someone who smoked cigarettes at the time of the interview. Current smokers comprised both daily smokers and occasional smokers (those who smoked on some days). Smoking purchasing behaviors were measured by several aspects. The first aspect was the price of cigarettes used, which was measured by a question, “How much money is each packet (20 manufactured cigarettes) of cigarettes you usually smoke?” Response options were less than 5 yuan/from 5 to less than 10 Yuan/from 10 to less than 15 Yuan/from 15 to less than 20 Yuan/20 Yuan or more. The second aspect was the quantity of cigarettes bought in a single purchase, which was measured by a question, “which way do you usually buy cigarettes” Response options were cartons /packs. The third aspect was source of cigarettes, which was measured by a question, “what was the main source of cigarettes used by you?” Response options were self-paying cigarettes/not self-paying (presented by others).

Environmental smoking restrictions included three aspects: households, public places, and work place. The measurement methodology is outlined by Yang et al. [13].

### City -level variables

There were several independent variables that reflected potential regional variation in the characteristics of the 21 study cities. The first was regional location, distinguished as east, central and west. The second aspect was population size and level of economic development (per capita Gross Domestic Product (GDP). The third aspect was cigarette production, which estimated using regional (province) cigarette production (pieces/100 Yuan GDP). The regional data were obtained from the National Bureau of Statistics [17]. The fourth aspect was tobacco control media news coverage, which was estimated by frequency of news per one million population. The news frequency was obtained by searching the Chinese research engine “Baidu” using four key words, “smoking” or “tobacco Control” or “banning on smoking restriction” or “smoking cessation” under “city name” in period of 2011 year” in the news category, which may reflect media news coverage level in each city for tobacco control. Each report obtained from news was scrutinized to ensure that it related to tobacco control, and all without tobacco control related content were excluded [18]. The fifth aspect covered environmental smoking restrictions in public places, workplaces and household, respectively with aggregating individual responses, categorized among cities by proportion of completed restriction [13]. We constructed contextual variables pertaining to each of the three environmental restriction categories based on aggregation of individual responses. The above variables were categorized, see Table 1.

**Table 1:** Demographics of sample and behavioral response prevalence

*Group*	N	% of sample	Decrease a little (%)	Significant decrease (%)	Unadjusted OR(overall change, PBR)	Unadjusted OR(considerable change, SPBR)
Individual level						
Age (years)						
<25	900	15.5	33.1	4.0	1.00	1.00
25–34	1333	20.4	33.3	4.6	1.04(0.80,1.35)	1.16(0.76,1.75)
35–44	1346	20.5	37.2	4.3	1.20(0.82,1.75)	1.09(0.48,2.46)
45–54	1335	19.6	39.2	4.5	1.30(0.97,1.75)*	1.13(0.59,2.16)
55+	946	24.0	35.5	6.5	1.27(0.88,1.83)	1.68(0.94,2.99)
*Gender*						
Male	5048	88.4	35.6	5.4	1.00	1.00
Female	612	11.6	37.5	0.9	0.84(0.39,1.82)	0.17(0.04,0.69)**
*Ethnicity*						
Han	5427	96.9	36.0	5.0	1.00	1.00
Other	233	3.1	29.4	0.8	0.60(0.35,1.04)*	0.15(0.04,0.50)***
*Education*						
Elementary school or less	1265	22.9	19.5	2.6	1.00	1.00
Junior high school	2823	46.9	40.5	4.8	2.85(1.17,6.90)***	1.84(0.81,4.20)
High school	825	16.4	33.4	5.9	2.31(0.83,6.41)	2.30(0.45,11.83)
Junior college or college	747	13.8	49.5	7.9	4.64(1.43,15.07)***	3.19(0.78,13.08)
*Occupation*						
Managers and clerks	977	15.1	17.3	1.3	1.00	1.00
Professionals	711	10.8	17.7	3.9	1.24(0.40,3.88)	0.30(0.47,19.83)
Commerce and service	1660	28.6	45.9	3.6	4.09(1.25,13.40)***	2.82(0.67,11.89)
Operations	1079	17.0	40.9	7.3	4.18(1.10,15.97)***	5.96(1.40,25.45)**
Students	445	13.0	41.1	9.3	4.69(1.22,18.05)***	7.69(1.34,44.33)**
Retired	222	3.3	37.3	5.4	3.37(0.96,11.87)*	4.29(0.64.28.76)
Other	566	12.2	37.8	5.1	3.31(0.88,12.56)	4.06(0.69,23.76)
*Income/person/year* Yuan*						
<10,000	2402	41.3	28.9	2.2	1.00	1.00
10,000-	1960	32.6	42.8	6.4	2.17(0.95,4.96)*	3.06(1.04,9.00)**
20,000-	1298	26.1	38.0	7.4	1.93(0.68,5.50)	3.59(0.96,13.43)*
*Type of smoking*						
Daily smoking	3753	66.9	24.1	2.4	1.00	1.00
Occasional smoking	1907	33.1	59.5	9.9	6.08(2.10,17.61)***	4.49(1.68,11.98)***
*Starting age for smoker*						
<16 year	867	14.9	3.5	1.0	1.00	
16-	1725	28.5	10.8	0.8	1.19(0.54,3.13)	0.89(0.37,2.13)
20-	3068	56.7	56.8	8.0	2.77(1.16,6.62)**	0.82(0.26,2.61)
*Cigarettes number*						
<10 cigarettes	1988	33.4	13.6	3.2	1.00	1.00
10 –cigarettes	1990	34.9	31.8	2.0	2.43(0.94,6.28)*	0.69(0.43,0.84)***
20- cigarettes	1682	31.7	63.6	9.8	12.46(2.94,52.80)***	3.28(0.69,16.07)
*Cigarette price*						
<5	818	15.3	5.1	5.1	1.00	1.00
5-	2342	40.1	21.8	1.8	2.58(0.78,8.47)	0.33(0.22,0.51)***
10-	1600	28.8	63.3	1.7	13.9(4.43,50.0)***	0.31(0.14,0.69)***
15-	506	8.7	76.8	6.7	31.3(5.4,79.0)***	1.32(0.16,11.62)
20-	394	7.2	48.8	36.8	42.5(6.9,97.6)***	10.80(1.55,75.49)**
Type for buy						
Cartons	3414	60.9	52.0	2.6	1.00	1.00
Packs	2146	39.1	16.0	9.2	0.36(0.15,0.75)***	0.27(0.13,0.57)***
*Smoking situation*						
Alone	1798	34.9	69.2	11.3	1.00	1.00
Smoking with others	3862	65.1	17.9	1.5	0.06(0.03,0.16)***	0.12(0.04,0.32)***
*Restrict smoking in public place*						
No or part	419	7.9	36.6	5.1	1.00	1.00
Restriction	5028	92.1	35.5	5.5	0.95(0.63,1.45)	0.98(0.62,1.58)
*Restrict smoking in household*						
No or part	3149	52.2	42.1	6.8	1.00	1.00
Restriction	2511	47.8	27.6	2.5	0.20(0.18,1.06)*	0.35(0.11,1.10)
*Restrict smoking in work place*						
No or part	2426	44.6	34.3	6.1	1.00	1.00
Restriction	3234	55.4	37.0	4.0	0.99(0.59,1.67)	0.64(0.26,1.58)
Regional variables						
*Region location*						
East	1913	45.9	37.9	6.6	1.00	1.00
Central	902	12.3	41.0	4.9	1.02(0.53,1.95)	0.72(0.30,1.77)
West	2845	41.8	31.9	2.9	0.65(0.32,1.33)	0.34(0.17,1.10)
*GDP* (yuan) *per capita*						
<40,000	2725	42.2	34.5	3.1	1.00	1.00
40,000-	1419	27.7	39.7	3.2	1.24(0.71,2.17)	1.03(0.58,1.82)
50,000-	2423	48.5	36.4	6.8	1.33(0.66,2.71)	2.27(0.94,5.43)*
*Population(million)*						
<5	2437	23.1	35.6	3.9	1.00	1.00
5-	2003	32.4	36.2	4.3	1.05(0.60,1.99)	1.10(0.55,2.20)
10 -	1220	44.4	35.5	5.9	1.54(0.60,3.96)	2.14(0.85,5.41)
*Cigarettes production*						
<5	1091	28.0	46.6	10.1	1.00	1.00
5-	1790	47.8	30.9	3.1	0.43(0.17,1.13)	0.29(0.12,0.71)***
10-	2490	32.7	42.8	3.5	0.70(0.27,1.80)	0.32(0.11,0.83)***
*Restrict smoking in public place*						
<70 %	2970	43.5	41.1	5.0	1.00	1.00
70 %-	1472	28.2	27.2	1.6	0.47(0.37,0.71)	031(0.19,0.48)
80 %-	1218	12.4	36.1	8.0	0.98(0.37,2.62)	1.65(0.62,4.39)
*Restrict smoking in household*						
<20 %	1192	23.1	26.8	1.9	1.00	1.00
25 %-	2270	44.8	41.0	6.9	2.35(1.27,4.35)***	3.86(1.58,9.53)***
35 %-	2148	32.1	35.0	4.2	1.62(0.85,3.08)	2.29(1.19,4.40)**
*Restrict smoking in work place*						
<30 %	1723	28.4	29.9	2.1	1.00	1.00
30 %-	2964	56.4	40.2	6.4	1.90(0.98,3.66)	3.15(1.42,6.93)***
40 %-	937	15.2	30.5	4.6	1.18(0.54,2.59)	2.54(1.05,6.63)**
*Medial news coverage*						
<20	4192	68.2	33.3	3.8	1.00	1.00
20-	589	7.9	42.3	2.6	1.31(0.69,2.52)	0.67(0.22,2.03)
40-	879	23.9	40.6	8.7	1.72(0.65,4.52)	2.59(1.05,7.20)**

*p < 0.1, **p < 0.05,***p < .0.01

### Data analysis

All data were entered into a database using Microsoft Excel (2010 version). The dataset was then imported into SAS (9.3 version) for the statistical analyses. Chi-square analyses were conducted using the SAS 9.3 survey procedure. Associations were confirmed through application of a multilevel logistic regression model using the SAS NLMIXED procedure [19].

Two multilevel logistic regression analyses were conducted. The first was to identify determinants of overall decreased smoking with cigarettes price, we operationalized our dependent variable, the price behavioral response (PBR), as an ordered response (no change = 1, decrease little = 2, decrease some or much = 3). The second was to identify determinants of much decreased smoking with cigarettes price, we operationalized our dependent variable, the strong price behavioral response (SPBR), as a binary response (no change or decrease little = 1, decrease some or much = 2). Both unadjusted and adjusted methods were considered in analyses. The unadjusted method was conducted though logistic regression using the SAS 9.3 survey procedure. The adjusted method was conducted through application of a multilevel logistic regression model using the SAS NLMIXED procedure [19],which added all of the possible confounders listed in Table 1 as covariates in the logistic models. For the first analysis an ordinal logistic multilevel model was used to estimate the effects for each explanatory variable by cumulative odds [20].

Two models were built for each analysis. The first was the ‘null’ model, a two-level model with random intercepts. A constant was the sole predictor for accounting for variation in the price behavioral response across 21 study cities. In this base model, we entered all demographic and regional level variables, as fixed main effects, to evaluate the separate impact of all individual-level and regional-level variables on the price behavioral response to form the full model. The independent variables in this analysis were those emerging as statistically significant (significant level: 0.1) in the unadjusted tests. All categories are listed in Table 1. The first category in each variable served as the referent in the logistic regression analysis. Backward stepwise regression is a preferred method for exploratory analyses, where analysis begins with a full or saturated model and variables are eliminated from the model in an iterative process. Model fitting was assessed by the likelihood of a change in the −2 log. Significance of the random parameter variance estimates was assessed using the Wald joint *X*^2^ test statistic [20].

All analyses were weighted. ^19^Weights included (1) sampling weights, as the inverse of the probability of selection, calculated at region, city, district, and community (2) Non-response weights consisted of household and individual aspects (3), and post-stratification weights were made by the combination of sex (male, female) and age (<25 year, 25– 35, 45, 55 and more) based on estimated distributions of these characteristics from a national survey [21]. The final overall subject-level weights were computed as the product of the above three weights. Chi-square analyses were weighted using the overall subject-level weights and the multilevel analyses were weighted using city level and subject-level weights respectively [19]. As these are no weight statement available the NLMIXED procedure the analysis was weighted though a macro method in this study [19].

## Results

A total of 18,875 individuals were identified as potential subjects for this study, among whom, 17,124 were effectively contacted and agreed to participate in the survey. Of the 17,124 surveys, 16,866 were valid questionnaires and utilized in this study. Of the respondents, 5660 were current smokers. Smoking prevalence was 30.6 % (95 % C.I:27.6 %–33.5 %). Turning to year to start smoking the mean was 20.78 (95 % C.I:19.64–21.92) for daily smokers, and 24.16 (95 % C.I:22.75–25.57) for occasional smokers. Smoking situation: more smokers smoked with others and 34.9 % (95 % C.I:30.6 %–39.2 %) smoked alone. More smokers thought their smoking to be no-addictive and 39.7 % (95 % C.I:34.5 %–44.1 %) thought their smoking to be addictive. Price of each box cigarettes used: 15.3 % (95 % C.I:10.7 %–19.9 %) of them were less than 5 yuan, 40.1 % (95 % C.I:36.0 %–44.2 %) were from 5 to less than 10 Yuan, 28.8 % (95 % C.I:24.8 %–32.8 %) were from 10 to less than15 Yuan, 8.6 % (95 % C.I:6.5 %–10.8 %) were from 15 to less than 20 Yuan, and 7.1 % (95 % C.I:5.7 %, 8.7 %) were 20 and more Yuan. Source of Cigarette used by smokers: most of them used self-paying cigarettes and 5.9 % (95 % C.I:4.8 %, 7.0 %) used cigarettes presented by others. Types of purchasing cigarettes: 67.2 % (95 % C.I:33.3 %, 71.1 %) of them buy in cartons, 32.8 % (95 % C.I:25.1 %, 40.5 %) in packs.

For the price behavioral response 56.9 % (95 % C.I:48.1 %, 65.7 %) of them indicated not change, 37.9 % (95 % C.I:31.0 %, 44.6 %) indicated decreased a little and 5.2 % (95 % C.I:2.6 %, 7.8 %) indicated decreased some or more. Table 1 shows unadjusted analyses results, PBR are significantly differences in individual age, ethnicity, education attainment,occupation, income, starting age for smokers, cigarettes number, type of smoking, cigarette price, types of purchasing cigarettes, smoking situation, and regional restriction in smoking household, while SPBR are significantly different in individual gender, ethnicity, occupation, income, cigarettes number, type of smoking, cigarette price, types of purchasing cigarettes, smoking situation, and regional per capita GDP, cigarettes production, restriction in smoking household, workplace and public place, medial news coverage. Table 2 shows the estimates generated in the multilevel analyses. For the former, full model analysis showed that an individual being Han, higher education attainment, occasional smoking, more cigarettes number and cigarette price, and smoking alone associated with higher the smoking expenditure decreasing. For the latter, male, Han, operations and students, occasional smoking, smoking alone, regional restrict smoking in work place and medial news coverage are associated with higher smoking expenditure decreases, but higher regional cigarette production is associated with a lower smoking expenditure decrease.

**Table 2:** Results of multiple level analyses

Group	PBR		SPBR	
Model	Null Model (OR, 95 % C.I)	Full Model 2 (OR, 95 % C.I)	Null Model (OR, 95 % C.I)	Full Model 2 (OR, 95 % C.I)
Individual level				
*Gender*				
Male				1.00
Female				0.17(0.03,0.94)**
*Ethnicity*				
Han		1.00		1.00
Other		0.41(0.29,0.58)***		0.18(0.05,0.70)***
*Education*				
Elementary school or less		1.00		
Junior high school		0.95(0.44,2.07)		
High school		1.06(0.24,4.64)		
Junior college or college		3.03(1.06,7.54)**		
Managers and clerks				1.00
Professionals				1.81(0.49,6.66)
Commerce and service				1.92(0.50,7.37)
Operations				4.30(1.11,16.66)**
Students				3.87(1.01,14.77)**
Retired				2.73(0.62,12.05)
Others				2.17(0.57,8.28)
*Smoking types*				
Daily smoking		1.00		1.00
Occasional smoking		3.37(1.28,7.88)***		1.98(1.20,3.25)**
*Cigarettes number*				
<10 cigarettes		1.00		
10 –cigarettes		2.11(1.06,4.18)**		
20- cigarettes		2.25(1.02,5.16)**		
*Smoking situation*				
Alone		1.00		1.00
Smoking with others		0.17(0.10,0.33)***		0.11(0.05,0.25)***
*Cigarette price*				
<5		1.00		
5-		1.46(0.56,3.78)		
10-		2.82(1.20,6.63)**		
15-		4.63(1.61,13.26)***		
20-		14.94(3.08,72.56)***		
Regional variables				
*Restrict smoking in work place*				
<30 %				1.00
30 %-				0.86(0.39,1.91)
40 %-				3.30(1.31,8.29)***
*Cigarettes production*				
<5				1.00
5-				0.15(0.09,0.26)***
10-				0.24(0.13, 0.46)***
*Medial news coverage*				
<20				1.00
20-				2.46(0.57,10.60)
40-				3.35(1.75,6.42)**
Random parameters between regions	V0:0.07 V1:0.05 Co1:0.02	V0:0.06 V1:0.02 Co1:0.02	2.81**	1.78
Fixed parameters	8.51***	4.40***	15.45***	4.28***

**p < 0.05,***p < .0.01

## Discussion

This study suggests smoking prevalence was 30.6 % (27.6 %–33.5 %). This study found most smokers used self-paying cigarettes, and 5.9 % used cigarettes given to them by others. This phenomenon is special in China culture. It is common to give cigarettes to guests or friends or to give cigarettes as gifts for services received [22]. Our analysis found the prevalence of not self-paying cigarettes is higher among professionals than other types of occupational groups. This may be because professionals provide valuable technical services and resources, and cigarettes are given to them in return for their services. Consequently, professionals are less sensitive to cigarette price, which may minimize the impact of increased cigarette price on consumption.

This study also found 67 % of smokers bought cigarettes in cartons, which is higher than reported by earlier researchers [23]. This indicates that currently the cigarette sales system in China is very convenient for buying cigarettes, and people can easily purchase cigarettes in the community, at work, or other places [15]. They choose to buy in cartons because they pay significantly lower prices than when purchasing in packs. This indicates that some consumers have to consider the question of price for cigarettes consumption.

This study shows 37.9 % of smokers indicated a little decrease in usage with the current cigarette price, and only 5.2 % of smokers decreased consumption some or more extent. This may be due to both market and consumer forces [1, 24]. This indicates that at the current retail price level smokers do not feel enough financial pressure when buying cigarettes to lead to a change their behavior [16], while another possibility is that Chinese smokers was not be enough sensitive enough to the overall price, which is consistent with Huang,s report [7]. Huang and his colleagues found that Cigarette consumption among low-income smokers did not decrease after a price increase, they reported that relative to other low-income and middle-income countries, cigarette consumption among Chinese adult smokers is not very sensitive to changes in cigarette prices [7]. Of course, this is a very complex phenomenon, many factors may contribute this phenomenon, the price-reducing behaviors, such as brand switching, trading down, and cigarette smuggling, and so on should be taken into account.

This study found that the random effects between-cities components in the null model were not significant but individual level variation test was significant in PBR analysis. This indicates that the cigarette consumption decreasing related to the price are influenced by individual variables, not environmental variables. After adding individual-level variables to form the null model for estimating the final model the fixed effect declined but retained its significance, which indicated some individual variables included can partly explain variations, but there are some potential individual variables that need to be explored. Different from PBR analysis, the random effects between-cities component in the null model were significant in SPBR analysis, which indicated there are significant differences in inter-city variation in this price response, and it was not significant in full model. This indicates some environmental variables included in this analysis can explain variations between-cities. Similarly the individual level variation test was significant in the null model, the fixed effect declined but retained its significance, which indicated some individual variables included in this analysis can partly explain variations, but there are some potential individual variables that need to be explored.

We noted some striking correlates of the price behavioral response. Whether PBR or SPBR minority groups were less sensitive than in the Han, which is consistent with studies about smoking behavioral, this difference may lie in their health awareness [10, 13, 16]. Smoking is a male norm and predominant practice in China, but this study found that males have more sensitive SPBR than females,which is not consistent with other studies [10, 25]. Our research underscores the importance of cultural norms in the diffusion of smoking and calls attention both to an opportunity and imperative to prevent the spread among Chinese females. This study found those with higher education attainment associated with higher PBR, when facing their perceived higher cigarette prices. A plausible reason is that they have greater health awareness in general as well as better knowledge of the health risks of tobacco smoking and advanced quitting skills [26, 27]. The SPBR associated with occupation status, which operations and students more sensitive to the price than reference. This may be because they have lower affordability for cigarettes due to their lower economic level [13, 24]. However, unlike other studies about cigarette consumption, also not consistent with economic theory: A dults with lower income are more price-responsive than adults with higher income [25, 28], income was not associated with price sensitivity in this study, but it was consistent with another a study from China [7]. PBR and SPBR were positively associated with occasional smoking, apparently this is related to nicotine dependence, where higher nicotine dependence prevents behavior change [14, 26]. Occasional smokers have lower nicotine dependence than daily smokers so they can easily change their behavior. This study found that more cigarettes number and higher cigarette price used associated with higher PBR, apparently, this phenomenon relates to the burden from cigarette consumption, which the demand for cigarettes was influenced by cigarettes price and the consumption amount [24]. About two-third (65.1 %) of current smokers in the study were found to smoke with other and this was associated with lower PBR and SPBR. This may be reason that social situations and peer influences are important smoking reasons and barriers to smoking behavior change in this Chinese population [27].

Our study found that that regional cigarette production is associated with the price sensitivity. This is unsurprising for many reasons, including the focus on regional economic development by government at the expense of addressing smoking problems or instituting tobacco control [1, 15], and the adversarial role of the tobacco industry as manifested in tobacco advertising and strong resistance to tobacco control measures [1, 29]. Currently in China, smokers and the tobacco industry are routinely depicted in a variety of overwhelmingly negative ways in everyday discourse and through the mass media. Tobacco advertising, promotion, sponsorship, and marketing are omnipresent in areas with heavy tobacco cultivation and cigarette production [1]. Tobacco companies also target consumers and potential consumers with culturally tailored cigarette brands [13]. These may make smokers less sensitive to the price.

Some studies found that news media coverage was associated with smoking behaviors and SHS exposure [11, 30]. This study is first to provide evidence about the influence of regional media coverage on the price sensitivity in a multilevel framework. This result suggest that the news media may influence people’s belief, awareness, and behaviors about smoking and tobacco control to make smokers more sensitive to cigarettes price. In common with findings for many other middle income and poorer countries, the overall public awareness of the hazards of smoking in China is low. This lack of awareness induces lower price sensitivity. Using the mass media is a powerful means for disseminating information about the adverse health effects of smoking to the general public and smokers, as reinforced by the regional findings of this study. Thus, a key challenge for China is to fortify efforts to harness the power and influence of the media to disseminate tobacco control messages, especially when they help to diminish the effects of tobacco advertising upon smoking.

Exposure to tobacco smoke in the workplace is correlated with increased risk for heart disease and lung cancer among adult nonsmokers [31]. Smoke-free workplaces not only protect nonsmokers from deleterious effects, but also decrease smoking prevalence [11, 32]. In our study, workplace restrictions were associated with the price sensitivity at the city-level. This finding likely reflects social norm change, and improvements in tobacco control awareness and beliefs due to the introduction of smoke-free workplaces which leads to more sensitivity to cigarettes price [13, 33, 34].

This study provided new information about a profile of price sensitivity, with several individual-level and regional variables contributing to smokers’ response behaviors to the price of cigarettes. This means that price sensitivity can be modified by changing the conditions conducive to smoking. We contend that although Chinese smokers appear less sensitive to cigarette prices than those in many other countries, this relationship is amenable to amelioration. Rarely have studies of the economics of smoking consumption considered price sensitivity to be modifiable. This study explored price sensitivity from an economic behavioral perspective. Our principal message is that price sensitivity is not just a problem at the individual level, but it also has an environmental context. Intervening at the regional level, through implementing workplace smoking restrictions, curbing cigarette production, and using news coverage may be able to change the price sensitivity of smokers. It is important to understand the price sensitivity issue, which has important implications for tobacco control policy in China. Increasing smoker sensitivity to cigarette prices will require stronger tobacco control and public education campaigns.

An important limitation of our study is its cross-sectional study design. Therefore, we cannot infer a causal link between these variables and price sensitivity. However, our study employed a large sample, and our findings satisfy several criteria for assuming causal inference, including strength of some associations, their consistency, and plausibility of effect. Future studies need to compile longitudinal surveillance data on cigarette price sensitivity. A second limitation is that only urban residents were included in our survey. Thus, our results are not generalizable to the overall population of China, which has a very substantial rural component. Thirdly, media coverage is reflected by Baidu search results, which could be biased as public service advertisements on television are not in Baidu. Third, we only concerned the behavioral response toward cigarettes price among smokers in this study, prior smokers (smokers quitted) did not include. So that our measure for the price behavioral response did not have quitting as an option. Further study should get information from both smokers and the quitters to fully understand the price sensitivity of cigarettes.

## Conclusions

This study adds substantially to the understanding of the behavioral responses of Chinese smokers to cigarette prices. It will be necessary to implement stronger tobacco control and public educational campaigns, enforce bans on smoking in workplaces, and regulate tobacco marketing practices in order to raise the price sensitivity of smokers.
