# Erratum to: Chinese smokers’ behavioral response toward cigarette price: individual and regional correlates

**DOI:** 10.1186/s12971-016-0099-2

**Published:** 2016-09-29

**Authors:** Tingzhong Yang, Sihui Peng, Lingwei Yu, Shuhan Jiang, William B. Stroube, Randall R. Cottrell, Ian R. H. Rockett

**Affiliations:** 1 Center for Tobacco Control Research, Zhejiang University School of Medicine, Yuhangtang Road, Hangzhou, 310058 China; 2 Health Services Administration Program, University of Evansville, Evansville, IN 47722 USA; 3 Public Health Studies Program, School of Health and Applied Human Sciences, University of North Carolina, Wilmington, NC 28403 USA; 4 Department of Epidemiology, School of Public Health, and Injury Control Research Center, West Virginia University, Morgantown, WV 26506-9190 USA

Following publication of the article in *Tobacco Induced Diseases* [1], we were made aware that the name of William B. Stroube was incorrectly captured in the article as William B. Stroub. The corrected author list can be seen above.

We apologize for the inconvenience this may have caused.
